# Comparative study of patients with chronic hepatitis C virus infection due to genotypes 1 and 3 referred for treatment in southeast Brazil

**DOI:** 10.1186/1471-2334-8-164

**Published:** 2008-12-04

**Authors:** Aline G Vigani, Maria H Pavan, Raquel Tozzo, Eduardo SL Gonçales, Adriana Feltrin, Viviane C Fais, Maria SK Lazarini, Neiva SL Gonçales, Fernando L Gonçales

**Affiliations:** 1 Grupo de Estudos das Hepatites Virais, Departamento de Clínica Médica, Faculdade de Ciências Médicas, Universidade Estadual de Campinas – UNICAMP, Sau Paulo, Brasil

## Abstract

**Background:**

The progression of liver disease in patients with chronic hepatitis C virus (HCV) infection is influenced by host and viral factors. Distinct clinical outcomes in patients infected with different HCV genotypes have been described in the literatute. However, the association between specific HCV genotype and clinical outcome remains unclear. We set out to study the natural history of HCV genotype 1 and 3 infections in Campinas, São Paulo state, Brazil, focusing on epidemiological, clinical, biochemical, and histological characteristics.

**Methods:**

Patients with HCV infection referred for treatment between January 2003 and December 2006 were included in this study. We collected epidemiological, clinical, and laboratorial data using standard forms.

**Results:**

A total of 283 patients were included; genotype 1 was idenfied in 163 (57.6%) patients, genotype 3 in 112 (39.6%), genotype 2 in 7 (2.5%), and genotype 4 in 1 (0.35%). Patients with genotype 2 and 4 were excluded from analysis. Multivariate analysis showed that intravenous energetic drug, positive cryoglobulin, and cirrhosis were independently and significantly associated with HCV genotype 3 (p < 0.05).

**Conclusion:**

Genotype 3 currently seems to be associated with intravenous energetic drug, high frequency of cryoglobulinemia, and advanced liver disease in our region. Understanding the distribution of the different HCV genotypes can elucidate transmission of HCV and support optimal prevention strategies.

## Background

Hepatitis C virus (HCV) infection affects approximately 170 million persons worldwide [1]. In some countries, such as the United States and some in west Europe, HCV is the main etiological factor of chronic hepatitis, end-stage cirrhosis, and hepatocellular carcinoma (HCC) [2,3]. The most common modes of HCV transmission is through percutaneous exposure to contaminated blood, transfusion of blood products before 1992, and intravenous drug use. Other potential risk factors for hepatitis C include intranasal use of cocaine, tattoing, body piercing, accidental needle-stick injury, sexual contact, and perinatal exposure. In Brazil, HCV infection was also associated with intravenous use of energetic drugs, such as Gluconergan^®^, a non-illicit drug commonly used in the 1970s as a stimulant [4,5].

The transmission of HCV is declining worldwide as a result of the screening of blood products and implementation of standard precautions [1]. However, the frequency of complications of HCV infection, including hepatic decompensation and HCC, are increasing and are expected to peak in 2015, because it can take several years for HCV-infection to progress to liver disease [1].

HCV has a heterogeneous natural history. After acute infection, 70% to 85% of patients develop chronic infection. Among those with chronic infection, 20% to 30% develop progressive fibrosis, cirrhosis, and other complications, such as portal hypertension and HCC after an average period of 20 years [6]. Conversely, chronic HCV infection might not cause considerable severe complications. Host and viral factors probably influence liver disease progression [7].

Comparison of nucleotide sequence data led to the classification of HCV into six distinct major genotypes whose genetic makeup differs by approximately 30% [8]. Genotype is closely associated with response to interferon (IFN) and ribavirin (RBV) therapy, but its influence on the course from HCV infection to liver disease remains unknown [7,9].

The geographic distribution of HCV genotypes is markedly heterogeneous throughout the world [10]. In addition, distinct epidemiological characteristic, clinical presentation, and histological outcome of cases caused by specific HCV genotypes have been described [11]. We evaluated the natural history of HCV genotype 1 and 3 infections in Campinas, São Paulo state, Brazil.

## Methods

We followed patients positive for HCV antibodies (Abbott AxSYM^® ^Anti-HCV 3.0, Abbott Laboratories, Wiesbaden, Germany) and positive for HCV RNA (Amplicor HCV 2^®^, Roche Diagnostics Systems Inc, Branchburg, USA) who presented at the teaching Hospital of the Universidade Estadual de Campinas, São Paulo state, Brazil from January 2003 through December 2006. This hospital is a regional reference center for treatment of hepatic disease. Patients already being treated with interferon or with concurrent infections caused by hepatitis B virus or HIV were excluded from this study. Patients were followed for at least six months after the completing hepatitis C treatment.

We collected relevant information on standard forms. Possible associations between HCV genotype and gender, age, source of transmission, aminotransferase levels, development of fibrosis, and cryoglobulinemia were investigated. Cryoglobulinemia was evaluated by an in-house method involving centrifugation and separation of 10 mL of serum and freezing at 0°C for seven days. All tests and liver biopsies were done on samples drawn prior to initiation of treatment.

We used amplicons generated by the Amplicor^® ^HCV test using a commercially available assay (Line Probe assay, LIPA HCV, Innogenetics, Gent, Belgium) to identify HCV genotype. This test is a reverse amplification assay based on the hybridization of the amplified sequence from 5'-nonconding region of HCV genome with oligonucleotide probes immobilized as parallel lines on membrane strips. The probes correspond to the sequence of the six most common HCV genotypes and their subtypes.

The diagnosis of cirrhosis was based on clinical and laboratory parameters (hyperbilirrubinemia, esophageal varices, ascites, splenomegaly), or histological examination. Fibrosis stage was evaluated using METAVIR score, where normal liver is designated as F0 and cirrhosis as F4 [12].

Patients with HCV genotype 1 infection received pegylated interpheron (PEG-IFN) alfa-2a (180 μg) or 2b (1.5 μg/Kg) subcutaneously once a week for 48 weeks, while those with HCV genotype 3 received interpheron alpha 3 MU subcutaneously three times a week for 24 weeks. All patients also received RBV, 1,000 mg to 1,250 mg, acording to patient's weight. Only data on patients who completed the proposed treatment and follow up for at least sixo months were included in the analysis. Sustained virological response (SVR) was defined as negative HCV RNA six months after the end of treatment.

### Statistical analysis

We analyzed data using EPI-INFO version 5.00 (Centers for Disease Control and Prevention, Atlanta, USA). P values less than 0.05 were considered statistically significant. We used chi-square and Mann-Whitney statistical tests, as appropriate. Variables for which an association with HCV genotype was suspected (i.e., p value = 0.20) were included in a multivariate stepwise logistic regression model to assess for confounding.

## Results

A total of 283 patients were considered elegible for inclusion in this study. Genotype 1 was detected in 163 (57.6%) patients, genotype 3 in 112 (39.6%), genotype 2 in 7 (2.5%), and genotype 4 in 1 (0.35%). Given the very low frequency of genotypes 2 and 4, patients infected with these genotype of HCV were excluded from analysis.

Two hundred one (73%) of the 275 patients included in the study were men. The median age was 43 years (range: 17–69 years). There was no significant difference in gender and age between patients with genotype 1 and 3 infections (Table 1). The most common risk factors for HCV infection were blood transfusion before 1992 (present in 71 [25.8%] patients), intravenous energetic drug use (Gluconergan^®^) (53 [19.3%]) and, intravenous illicit drug use (44 [16.0%]). Eleven (4%) patients reported two risk factors. Eighty-nine (32.4%) patients did not have any HCV risk factor identified (Table 1). Blood transfusions before 1992 was found with greater frequency in genotype 1 (28%) rather than genotype 3 (21.4%) infections, but this difference was not statistically significant (p = 0.17). Previous use of Gluconergan^® ^was statistically significantly associated with genotype 3 (p = 0.008) (Table 1). The median alanine aminotransferase (ALT) and aspartate aminotransferase (AST) levels were higher in genotype 3 than genotype 1 (p = 0.003 and p = 0.0001, respectively) (Table 1). When we compared patients with and without detectable serum cryoglobulins, we found that cryoglobulinemia was more frequent among genotype 3-infected patients rather than those infected with genotype 1 infection (p = 0.001) (Table 1).

**Table 1:** Patients characteristics associated with hepatitis C virus (HCV) genotypes 1 and 3 infections (n = 275).

**Characteristic**	**Genotype 1 (n = 163)**	**Genotype 3 (n = 112)**	**p value**
	**n (%)**	**n (%)**	
Male	114 (69.9)	87 (77.7)	0.15
Age (median range, yr)	43.0 (25–69)	42.5 (17–67)	0.70
Risk factor for HCV infection*			
Intravenous energetic drug	23 (14.1)	30 (26.8)	**0.008**
Blood transfusion	47 (28.8)	24 (21.4)	0.17
Intravenous illicit drug use	22 (13.5)	22 (19.6)	0.17
Unknown risk	58 (34.5)	31(27.7)	0.16
Others**	18 (11.0)	11 (9.8)	0.75
ALT (median)	62.0	81.5	**0.003**
AST (median)	47.0	65.0	**0.001**
GT (median)	61.0	70.0	0.58
Positive cryoglobulin	88 (54.0)	80 (71.4)	**0.001**

*Eleven patients had two risk factors

** Others includes intranasal use of cocaine, tattoing, body piercing, accidental needle-stick injury

Reference ranges: ALT (alanine aminotransferase): 6–40 U/L; AST (aspartate aminotransferase): 6–40 U/L; 3/4GT (glutamyl transferase): 9–40 U/L

Liver biopsies were perfomed on 241 (89%) of 275 patients. An additional 21 (8.1%) patients had clinical signs and laboratory findings consistent with cirrhosis and consequently a liver biopsy was not perfomed. Thus, the analysis of stage of fibrosis was limited to 262 (95.3%) patients. Thirty-eight (15.8%) of the 241 patients had histopathologically confirmed cirrhosis and 21 had clinical cirrhosis (Table 2). Histologic findings of chronic hepatitis without cirrhosis were present in 203 (78.2%) patients. Severe fibrosis (F3, F4, and clinical cirrhosis) was more frequent among patients infected with genotype 3 (p = 0.0004) (Table 2). In addition, patients infected with genotype 3 had cirrhosis significantly more often compared with patients infected with genotype 1 (p = 0.001) (Table 2).

**Table 2:** Stage of fibrosis by Metavir system associated with HCV genotypes 1 and 3 in patients with chronic hepatitis C (n = 262).

**Stage of fibrosis**	**Genotype 1 (n = 154)**	**Genotype 3 (n = 108)**
	**n (%)**	**n (%)**
Mild or moderate	111 (72)	55 (51)
F0	4 (3)	0 (0.0)
F1	33 (21)	15 (14)
F2	74 (48)	40 (37)
Severe fibrosis	43 (28)	53 (49)
F3	19 (12)	18 (17)
F4 and clinical cirrhosis	24 (15.6)	35 (32.2)

We initiated treatment of disease in 180 (65.4%) patients. Among those, 39 (36.2%) of 97 patients with genotype 1 and 35 (45.5%) of 83 with genotype 3 achieved SVR. Multivariate stepwise logistic regression analysis showed that clinical or histological cirrhosis (adjusted odds ratio [OR] = 1.93, 95% confidence interval [CI] = 1.0–3.7), positive cryoglobulin (adjusted OR = 1,96; 95% CI = 1.11–3.46), and use of Gluconergan^® ^(adjusted OR = 2,0; 95% CI = 1.03–4.09) were independently associated with HCV genotype 3.

## Discussion

HCV genotypes 1, 2, and 3 have a worldwide distribution but their relative prevalence varies from one geographical area to another. In North America and Europe, genotype 1 is responsible for over 70% of all HCV infections followed by genotype 2 and then genotype 3 [9]. Genotype 4 is found almost exclusively in the Middle East and North Africa, while genotypes 5 and 6 are common in South Africa and Asia, respectively [9,13].

In agreement with previous studies in Brazil, we demonstrated that HCV genotype 1 was the most prevalent genotype, followed by genotypes 3 and 2 [4,14]. The previously reported prevalence of genotype 3 among patients with HCV infection in Brazil, around 30%, is among the highest in the world [14]. In our study, which was done among patients referred for treatment of hepatic disease, we found an even higher genotype 3 prevalence, 40%.

An association between HCV genotypes and the mode of HCV transmission has been reported by some authors in certain areas of world [9,15]. In Europe, intravenous illicit drug use is more common in patients with genotype 3 infection, while blood transfusion is commonly associated with genotype 1 [13]. However, in the United States, some authors did not find an association between HCV genotypes and modes of HCV transmission [9]. In our study, blood transfusion prior to 1992 was the most frequently reported risk factor for genotype 1 infection, while the transmission of genotype 3 was associated with intravenous energetic drug (Gluconergan ^®^) use. This finding is in agreement with other studies in different regions of Brazil [4,5]. Knowledge of the frequency and distribution of the HCV genotypes can provide information to better understand the transmission of HCV and direct the development of prevention strategies.

The association of HCV infection and cryoglobulinemia has been previously reported and may be associated to the virus' ability to infect mononuclear cells [16]. However, the association between HCV genotype and cryoglobulinemia remains unclear. Some studies have not found an association between HCV genotype and cryoglobulinemia, while others have found higher frequencies of cryoglobulinemia among individuals infected with genotype 1 or 2 infections [17-19]. The association between cryoglobulins and a specific genotype varies from one region to another and results from an interaction betwen viral and host factors. The high frequency of cryoglobulins in patients with genotype 3 infection observed in our study could have resulted from hight frequency of cirrhosis associated with this genotype, since crioglobulinemia may represent a complication in patients with HCV infection, especially in those with cirrhosis [16,20].

The rate of progression of fibrosis varies markedly among HCV-infected patients and the major factors known to be associated with fibrosis progression are older age at the time of infection, male gender, and excessive alcohol use [7]. There is little evidence that viral factors, such as genotype, play a role in severity and outcome of liver disease. In some studies from the United States and Europe, HCV genotype 1 was found to be associated with a higher frequency of cirrhosis [9,21]. However, many of these studies did not address possible confounding factors, such as age and duration of infection. In studies that addressed the confounding effect of these variables, the association between genotype 1 and more severe liver disease was not found [9,22].

We observed that patients infected with genotype 3 have a higher frequency of cirrhosis, higher ALT and AST levels, and the acquisition of HCV was related to intravenous energetic drug. Quite possibly patients infected with genotype 3 in our region may represent a sub population that has characteristics associated with a more severe liver disease, such as consumption of alcohol or the longer length of the infection. We were not able to analyze these factors as part of this study.

Previous studies have shown that patients with genotype 1 infection treated with PEG-IFN alfa-2a or 2b plus RBV achieved SVR rates of 42% to 46%; while patients with genotype 3 infection treated with interferon alfa plus RBV achieved SVR rates of 61% [22,23]. We found lower SVR rates for genotype 1 and 3 infections (36.2% and 45.5%, respectively) than these studies. A limitation of this study is that dose reduction and race are important predictors for SVR and we were unable to account for those. Further studies are needed to confirm our findings and explore the reasons for these different response rates, since this would help design treatment plans for infected patients.

## Conclusion

In conclusion, genotype 1 was found to be the main genotype in our study followed by genotype 3. Additionaly, genotype 3 currently seems to be associated with intravenous energetic drug, high frequency of cryoglobulinemia, and advanced liver disease in our region. It is important to consider and address these epidemiological and clinical differences since chronic hepatitis is likely to an extra burden to the healthcare system considering the cost and social consequences associated with advanced liver disease.

## Competing interests

The authors declare that they have no competing interests.

## Authors' contributions

AGV, participated in the design of the study, performed the statistical analysis, conceived of the study, and made substantial contributions to acquisition, analysis, and interpretation. MHP, RT, ESLG and MSKL made substantial contributions to acquisition, analysis, and interpretation of data. AFF perfomed liver biopsis and participated in the design of the study. VCF carried out the molecular genetic studies and drafted the manuscript. NSLG coordinated the laboratory analyses carried out the molecular genetic studies and drafted the manuscript. FLG Jr, have been involved in drafting the manuscript, revising it critically for important intellectual content and coordination. All authors read and approved the final manuscript.

## Pre-publication history

The pre-publication history for this paper can be accessed here:


